# Dorsomorphin, a Selective Small Molecule Inhibitor of BMP Signaling, Promotes Cardiomyogenesis in Embryonic Stem Cells

**DOI:** 10.1371/journal.pone.0002904

**Published:** 2008-08-06

**Authors:** Jijun Hao, Marie A. Daleo, Clare K. Murphy, Paul B. Yu, Joshua N. Ho, Jianyong Hu, Randall T. Peterson, Antonis K. Hatzopoulos, Charles C. Hong

**Affiliations:** 1 Division of Cardiovascular Medicine, Department of Medicine, Vanderbilt University School of Medicine, Nashville, Tennessee, United States of America; 2 Department of Pharmacology, Vanderbilt University School of Medicine, Nashville, Tennessee, United States of America; 3 Cardiovascular Research Center and Division of Cardiology, Department of Medicine, Massachusetts General Hospital, Harvard Medical School, Boston, Massachusetts, United States of America; 4 Department of Cell and Developmental Biology, Vanderbilt University School of Medicine, Nashville, Tennessee, United States of America; Katholieke Universiteit Leuven, Belgium

## Abstract

**Background:**

Pluripotent embryonic stem (ES) cells, which have the capacity to give rise to all tissue types in the body, show great promise as a versatile source of cells for regenerative therapy. However, the basic mechanisms of lineage specification of pluripotent stem cells are largely unknown, and generating sufficient quantities of desired cell types remains a formidable challenge. Small molecules, particularly those that modulate key developmental pathways like the bone morphogenetic protein (BMP) signaling cascade, hold promise as tools to study *in vitro* lineage specification and to direct differentiation of stem cells toward particular cell types.

**Methodology/ Principal Findings:**

We describe the use of dorsomorphin, a selective small molecule inhibitor of BMP signaling, to induce myocardial differentiation in mouse ES cells. Cardiac induction is very robust, increasing the yield of spontaneously beating cardiomyocytes by at least 20 fold. Dorsomorphin, unlike the endogenous BMP antagonist Noggin, robustly induces cardiomyogenesis when treatment is limited to the initial 24-hours of ES cell differentiation. Quantitative-PCR analyses of differentiating ES cells indicate that pharmacological inhibition of BMP signaling during the early critical stage promotes the development of the cardiomyocyte lineage, but reduces the differentiation of endothelial, smooth muscle, and hematopoietic cells.

**Conclusions/ Significance:**

Administration of a selective small molecule BMP inhibitor during the initial stages of ES cell differentiation substantially promotes the differentiation of primitive pluripotent cells toward the cardiomyocytic lineage, apparently at the expense of other mesodermal lineages. Small molecule modulators of developmental pathways like dorsomorphin could become versatile pharmacological tools for stem cell research and regenerative medicine.

## Introduction

Pluripotent stem cells, which are capable of self-renewal and differentiation into multiple tissue types, show enormous potential as a source of cells to repair damaged adult tissues [1], [2]. For example, replacement of damaged heart muscle with cells derived from pluripotent stem cells offers hope for improving the outcomes of millions of patients with heart failure, whose current treatments remain largely palliative. Recent advances in reprogramming adult somatic tissue to generate induced pluripotent stem (iPS) cells, which possess ES-like features, have heightened the expectation for successful regenerative therapies [3]–[7]. Nonetheless, numerous and formidable challenges must be overcome before the regenerative potential of stem cells can be fully harnessed. One such challenge is the development of reliable methods and tools for generating desired cell types from pluripotent cells.

*In vitro* differentiation of pluripotent ES cells provides an excellent framework for exploring the developmental programs of a number of distinct tissue types, including cardiac cells. Examining how ES cells differentiate into functioning cardiomyocytes *in vitro* may ultimately reveal strategies to augment the cardiogenic potential of pluripotent stem cells, including the iPS cells. While the mechanisms by which myocardial cells are generated from ES cells are still poorly understood, recent studies indicate that cardiomyogenesis occurs largely through a step-wise progression of lineage commitment [8], rather than simple induction of uncommitted cells by “cardiogenic” conditions [9]. Therefore, successful approaches to control and promote development of cardiomyocytes from stem cells will likely involve timely modulation of signaling pathways involved in embryonic cell-fate specification, such as bone morphogenetic protein (BMP) signaling [10].

While a variety of methods can be employed to regulate developmental pathways, selective small molecule modulators in particular may become valuable tools for directing differentiation of stem cells [11]–[13]. For example, a small molecule that can block the effects of multiple BMP ligand subtypes and receptors might be useful in contexts where the specific cocktail of BMPs and cognate BMP antagonists at play is difficult to pin point. Moreover, small molecules permit exquisite temporal control over BMP signaling. This might be particularly important for functional dissection of BMP signaling in complex biological settings like *in vitro* ES cell differentiation, where BMP signals are required at multiple time points to regulate a number of diverse developmental events [10], [12], [14]–[16].

In a chemical screen for small molecules that disrupt dorsoventral patterning in zebrafish embryos, we recently identified dorsomorphin (6-[4-(2-Piperidin-1-yl-ethoxy)phenyl]-3-pyridin-4-yl-pyrazolo[1,5-a]pyrimidine), also known as compound C [17], which selectively inhibits BMP type I receptors [18]. Since the natural BMP inhibitor Noggin has been shown to promote mouse ES cell differentiation into cardiomyocytes [10], we examined whether dorsomorphin could also enhance cardiomyogenesis. Here, we show that dorsomorphin treatment of mouse embryonic stem (ES) cells leads to a strong expansion of the cardiomyocytic lineage in a controlled manner. In contrast to cardiac induction by Noggin, which requires 5 days of treatment beginning at 3 days before the initiation of ES cell differentiation, dorsomorphin treatment limited to the first 24-hours of differentiation is sufficient for robust cardiac induction. Moreover, our results indicate that inhibition of BMP signaling during the initial stages of differentiation promotes cardiomyogenesis at the expense of endothelial, smooth muscle, and hematopoietic lineages.

## Results

### Small molecule BMP inhibitor, dorsomorphin, induces cardiomyogenesis in mouse ES cells

**Figure 1:**
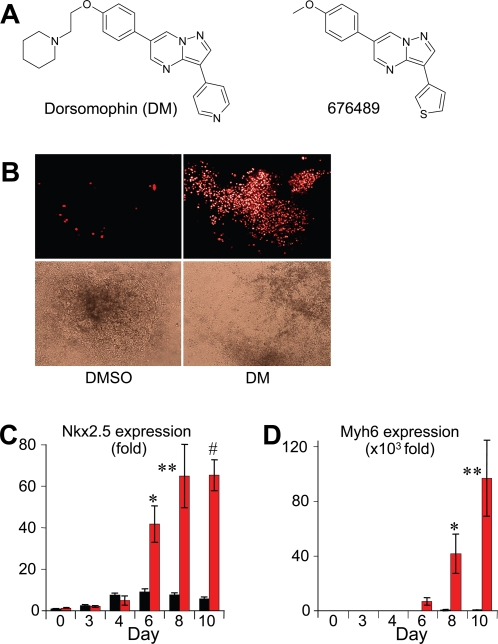
The small molecule BMP inhibitor, dorsomorphin, induces cardiomyogenesis in mouse ES cells. (A) Chemical structures of dorsomorphin (DM), a selective BMP inhibitor, and 676489, a DM analog which inhibits VEGF/VEGFR2, but not BMP signaling. (B) ES cells treated with dorsomorphin (DM) from day −3 to 2 formed large areas of contracting cardiomyocytes that expressed DsRed-Nuc under the α-MHC promoter by day 12 of differentiation (right), but DMSO-treated cells did not (left). Upper panels depict representative red fluorescence images. Lower panels show the corresponding bright-field images. (C, D) Dorsomorphin treatment resulted in strong increases in expression of cardiac markers Nkx2.5 (*p = 0.021, **p = 0.020, #p = 0.0013), and Myh6 (*p = 0.046, **p = 0.026). Q-PCR results represent relative expression normalized to that of DMSO-treated cells at Day 0. Measurements were from at least three independent experiments for each time-point.

To gauge cardiomyogenesis, we used the mouse ES cell line CGR8, which was stably transfected with a construct expressing the red fluorescent protein gene fused to a nuclear localization signal (DsRed-Nuc) under the alpha-myosin heavy chain (α–MHC) promoter [19]. In this system, α–MHC expressing cells are marked with red nuclear fluorescence, allowing a visual, quantitative assessment of differentiating cardiomyocytes. The cells were treated with 2 µM dorsomorphin (**Figure 1A**), which effectively blocks BMP-induced SMAD activation [20], but not AMP-activated kinase activity [17]. Based on observations by Yuasa et al. [10], dorsomorphin was administered 3 days prior (day −3) to the initiation of embryoid body (EB) formation. Dorsomorphin was added with daily changes of ES media until day 0, when EB formation was initiated in hanging drops containing EB/differentiation medium with an additional dose of dorsomorphin. At day 2 of EB formation, dorsomorphin was washed out. The dorsomorphin vehicle DMSO was used as negative control.

Under these conditions, dorsomorphin-treated αMHC-DsRed-CGR8 cells formed large, synchronously beating areas containing spontaneously contracting cells that expressed DsRed protein within 12 days of differentiation (**Figure 1B** **, Movie S1, Movie S2**). Increases in beating areas corresponded to a 20 to 30-fold increase in the frequency of cardiomyocytes, as indicated by DsRed-Nuc^+^ or α-actinin-staining cells (see below). Dorsomorphin-induced cardiomyogenesis was associated with large increases in expression of several cardiac genes, as measured by quantitative real-time PCR (Q-PCR). Compared to controls, dorsomorphin treatment increased expression of the early cardiac marker Nkx2.5 by 11.4-fold (**Figure 1C**), the cardiac myosin heavy chain gene (Myh6) by 125-fold (**Figure 1D**), and the cardiac myosin light chain 2 (Myl2) by 34.2-fold (**Figure S1F**) at day 10 of differentiation. The time course for robust Myh6 and Myl2 gene induction correlated closely with the appearance of DsRed+ cells starting at day 8 of differentiation, confirming that DsRed-Nuc, expressed by the stably transfected α-MHC promoter construct, is an accurate marker of cardiomyocytic differentiation. With regular changes in culture media, both the spontaneous contractions and the red fluorescence could be maintained for at least 4 weeks, suggesting that the observed cardiac phenotypes were the consequence of a permanent differentiation program rather than a transitory process.

Substantial increases in contracting areas were also noted with dorsomorphin treatment of unmodified CGR8 and R1 mouse ES cell lines maintained by three independent laboratories. Moreover, the structurally related compound 676489 (**Figure 1A**), a selective inhibitor of the type-2 vascular endothelial growth factor receptor type-2 (VEGFR2, Flk), which does not inhibit BMP signaling, did not induce cardiomyogenesis in ES cells under this condition or any of the additional conditions described below (data not shown). Thus, the cardiac induction by dorsomorphin is not restricted to the modified α-MHC-DsRed-Nuc expressing CGR8 cells, and likely mediated by inhibition of BMP signaling.

### A 96-well microtiter format for quantitative assessment of cardiac induction by dorsomorphin treatment

**Figure 2:**
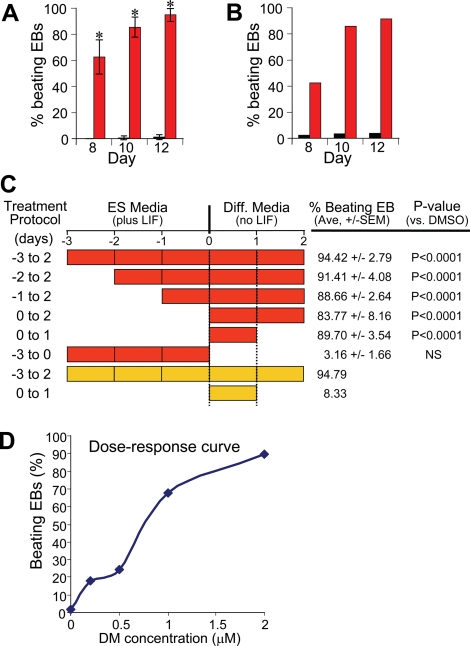
Quantitative assessment of cardiomyocyte induction by dorsomorphin in a 96-well format. (A) Dorsomorphin (DM) treatment in a 96-well microtiter plate format reproducibly induced formation of beating embryoid bodies in CGR8 ES cells (94.4% of EBs, *p<0.0001, results from at least three independent experiments involving over 300 EBs per condition). (B) DM treatment reproducibly induced cardiomyogenesis in R1 ES cells (91.3% of EBs, results from at least 92 EBs). All results are compared to DMSO-vehicle treatment as negative control. Red bars, dorsomorphin-treated. Black bars, DMSO-treated. Error bars represent standard error. (C) Time window for cardiomyocyte induction by dorsomorphin. DM treatments from Day −3 to 2, Day −2 to 2, Day −1 to 2, Day 0 to 2, and Day 0 to 1, represented by red bars, were nearly equivalent in promoting formation of beating cardiomyocytes in mouse ES cells at day 12 of differentiation. Increases in the frequencies of beating EBs with the above DM treatment protocols were all highly statistically significant in comparison to DMSO treatment over same time periods (P<0.0001 for each condition). Differences between the above DM treatments were not statistically significant. In contrast, DM treatment from Days −3 to 0 did not result in significant increase in cardiomyocyte formation compared to DMSO control. Based on these results, the minimal temporal requirement for cardiac induction by DM can be narrowed down to the first 24 hours of ES cell differentiation (shown between two dotted lines). Frequencies of DM-treated EBs that contract spontaneously by day 12 were obtained from at least 200 EBs for each time point on three or more separate days. Ave. denotes average percentage of EBs that contract spontaneously. By contrast, Noggin treatment (300 ng/mL), represented by orange bars, during the first 24 hours of differentiation did not efficiently induce the formation of beating EBs. However, in agreement with the prior report by Yuasa et al, Noggin treatment from Days −3 to 2 (orange bars) efficiently induced the formation of beating EBs. For Noggin treatment, results were obtained from 96 EBs per condition. (D) Dose-response curve for cardiomyocyte induction by DM treatment from Day 0 to 1. ES cells were treated with various concentrations of DM at day 0 to 1 of differentiation, and percentages of EBs that contract spontaneously at day 12 of differentiation were determined. Results were then used to create a dose-response curve. Results were obtained from at least 52 EBs per condition.

A major challenge to studying *in vitro* cardiomyocyte development in ES cell models is the considerable variation in cardiomyogenesis efficiency under different culture conditions. For example, we have found that, even in the absence of specific chemical or molecular manipulation, the frequency of formation of spontaneously contracting areas could vary substantially, depending on FBS concentration and density of EBs plated per well as well as stochastic events. To assess the impact of dorsomorphin treatment in a more reproducible, and quantitative manner, EB formation was initiated in 96-well microtiter plates. Using this technique, aliquots of 500 ES cells were distributed in uncoated round bottom microtiter plates in differentiation media, and cells were allowed to aggregate at the bottom of each well by gravity or by brief centrifugation. Any EB that contained visible clusters of spontaneously contracting cells was recorded as 1 positive well. Using this method, a reproducible average of about 1 to 2% of DMSO-treated EBs were found to contract by day 12 of differentiation (**Figure 2A** **, Movie S3**). By contrast, 94.4% of EBs treated with 2 µM dorsomorphin from day −3 to ay 2 contracted spontaneously by day 12 (**Figure 2A**). Dorsomorphin was also effective at robustly inducing cardiomyogenesis in unmodified CGR8 and R1 mouse ES cell lines, indicating that the procardiogenic effects of dorsomorphin under this condition were not cell line-restricted (**Figure 2B**).

### Dorsomorphin treatment limited to the first 24-hours of ES cell differentiation is sufficient for robust cardiac induction

The 96-well microtiter format permitted quantitative assessments of cardiac induction by different dorsomorphin treatment protocols. Using this method, we determined the critical time window for the dorsomorphin effect. We found that treatment starting at day −2 (day −2 to 2), day −1 (day −1 to 2) and day 0 (day 0 to 2) were nearly as effective in promoting cardiac induction as the day −3 to 2 protocol (beating frequencies ranging from 83.8 to 94.4%, **Figure 2C**), but dorsomorphin treatment from day −3 to 0 resulted in only 3.2% beating frequency (**Figure 2C**). Importantly, dorsomorphin treatment begun at the time of EB formation for just 24 hours (day 0 to 1) was highly effective in promoting cardiomyogenesis, displaying beating frequencies of 89.7% (**Figure 2C**). Thus, the minimum time window for cardiac induction by dorsomorphin lies within the first 24 hours of ES cell differentiation. By contrast, in line with the previously reported results with Noggin [10], robust cardiac induction was observed only with Noggin treatment from day −3 to 2, but not with Noggin treatment from day 0 to 1 of differentiation (**Figure 2C**). Finally, a dose-response relationship for dorsomorphin-induced cardiomyogenesis was determined using the 96-well EB differentiation format (**Figure 2D**).

**Figure 3:**
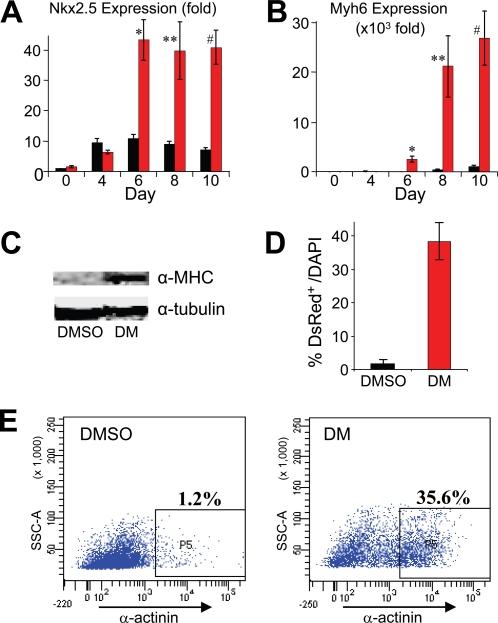
Dorsomorphin treatment during the initial 24 hours of ES cell differentiation robustly induces cardiomyogenesis. (A, B) Dorsomorphin (DM) treatment from day 0 to 1 of differentiation resulted in strong increases in expression of cardiac markers Nkx2.5 (*p = 0.003, **p = 0.0019, #p = 0.0001), and Myh6 (*p = 0.023, **p = 0.029, #p = 0.0089). All results are compared to DMSO-vehicle treatment as negative control. Red bars, DM-treated. Black bars, DMSO-treated. Error bars represent standard error. Q-PCR results represent relative expression normalized to that of DMSO-treated cells at day 0. Measurements were obtained from at least three independent experiments for each time-point. (C) Western blot showing markedly higher levels of the cardiac α-Myosin Heavy Chain (α-MHC) protein in DM-treated ES cells on day 10 in comparison to DMSO-treated controls. Antibody against α-Tubulin was used as loading control. (D) DM treatment greatly increased the number of fluorescent nuclei that expressed DsRed as a percentage of total cells (DAPI+) at day 12 (error bars, standard error; p<0.0001 vs. DMSO control). Results from trypsin dissociated cells from 11 DM-treated EBs and 14 DMSO-treated controls. (E) Representative FACS analysis showing an approximately 30-fold increase in the fraction of α-actinin+ cells following DM treatment vs. DMSO controls.

Dorsomorphin treatment from day 0 to 1 of differentiation, which resulted in a 96.1% decrease in the BMP-response Id1 expression [21] in comparison to DMSO control (**Figure S2**), led to substantial increases in contacting areas, in comparison to DMSO controls. These increases were reflected in greater areas that immunostained for the cardiac-specific transcription factor Nkx2.5, and the sarcomeric proteins α-Actinin, cardiac Troponin-T and cardiac α-MHC (see below). Moreover, as observed for the day −3 to 2 treatment, the 24-hour dorsomorphin treatment was associated with very large increases in the expression of cardiac-specific genes Nkx2.5 and Myh6, as measured by Q-PCR (up to 5.8-fold and 100-fold induction, respectively; **Figure 3A, B**). Consistent with both the immunostaining and Q-PCR results, western blots with cardiac α-MHC antibody showed markedly higher cardiac α-MHC protein levels in dorsomorphin-treated EBs than in controls (**Figure 3C**).

Cardiac induction by dorsomorphin treatment during the initial 24 hours of differentiation was quantified by two distinct approaches using two ES cell lines. In the first, day 12 EBs from α-MHC-DsRed CGR8 cells were dissociated with trypsin and stained with DAPI, followed by microscopic examination to count the total number of cells (DAPI+) and the DsRed+ cardiomyocytes. Using this method, an average of 38.4% of dorsomorphin-treated cells were DsRed+, while 1.8% of DMSO-treated cells were DsRed+, representing an approximately 21-fold increase in the relative abundance of cardiomyocytes with dorsomorphin treatment (**Figure 3D**). Next, the cardiomyocyte induction in the parental CGR8 cell lines was measured by FACS analysis. On day 12, EBs treated with dorsomorphin or DMSO were dissociated, and stained with anti-α-Actinin and secondary AlexaFluor-488 antibodies. FACS analyses showed that approximately 35 to 40% of dorsomorphin-treated ES cells were positive for sarcomeric protein α-Actinin, whereas only 1 to 2% of DMSO-treated cells were positive for α-Actinin, representing an approximately 30-fold induction in frequency of α-Actinin+ cells (**Figure 3E**).

### Characterization of cardiomyocytes induced by dorsomorphin treatment

**Figure 4:**
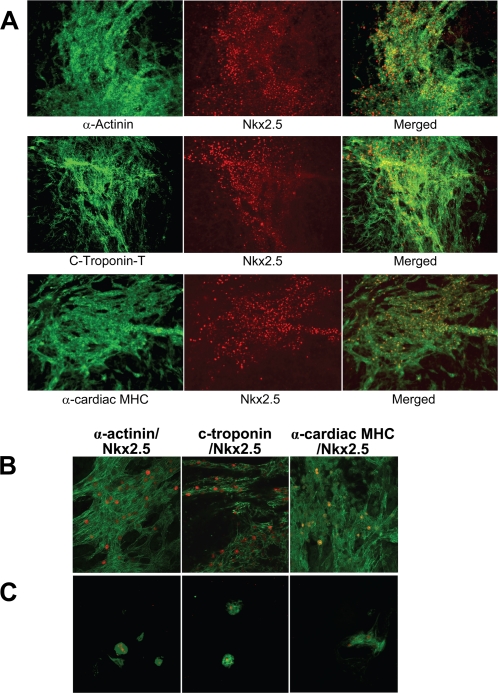
Dorsomorphin treatment promotes the formation of large areas comprised of cardiomyocytes. (A) Dorsomorphin-treated ES cells formed larger areas of contracting cardiomyocytes that immunostained for the sarcomeric proteins cardiac Troponin-T (c-TnT), α-Actinin and α-cardiac myosin heavy chain (α-MHC) at day 10 (Green, left panels). The areas that immunostained for sarcomeric proteins also immunostained for the cardiac-specific transcription factor Nkx2.5 (Red, middle panels), confirming that these regions are comprised mainly of cardiomyocytes. Merged images are on the right. (B) Merged 40× confocal images showing the details of sarcomeric protein and Nkx2.5 immunostaining (right, α-actinin/Nkx2.5; middle, c-troponin-T/Nkx2.5; right, α-cardiac MHC/Nkx2.5). DM-treated cells showed many cardiomyocytes with organized sarcomeric structures. (C) 10× images of DMSO-treated cells. In control conditions, cells that immunostain for sarcomeric proteins and Nkx2.5 are rare and typically form much smaller foci without discernable sarcomeric organization. The panels depict representative immunofluorescence images.

To confirm that the large synchronously contracting areas of DsRed+ cells induced by the dorsomorphin treatment were composed of cardiomyocytes, dorsomorphin EBs were fixed at day 12 and processed for immunostaining with specific antibodies against several known cardiac markers. In dorsomorphin-treated EBs, large areas that immunostained for the sarcomeric proteins α-Actinin, cardiac Troponin-T (c-TnT), and cardiac α-Myosin Heavy Chain (α-MHC) are readily observed (**Figure 4A, B**). Moreover, Nkx2.5 immunostaining colocalized with α-Actinin, c-TnT, and α-MHC+ areas, confirming that large patches of cardiomyocytes were induced by dorsomorphin treatment (**Figure 4A, B**). Confocal microscopy confirmed the sarcomeric organization of α-Actinin, c-TnT, and α-MHC staining in dorsomorphin-induced cardiomyocytes (**Figure 4B**). By contrast, in control EBs, areas that immunostained for α-Actinin, c-TnT, and α-MHC were rare, and foci comprised of few isolated cells, lacking sarcomeric organization (**Figure 4C**).

### Pharmacological BMP inhibition promotes cardiomyogenesis at the expense of other mesoderm-derived cell lineages

**Figure 5:**
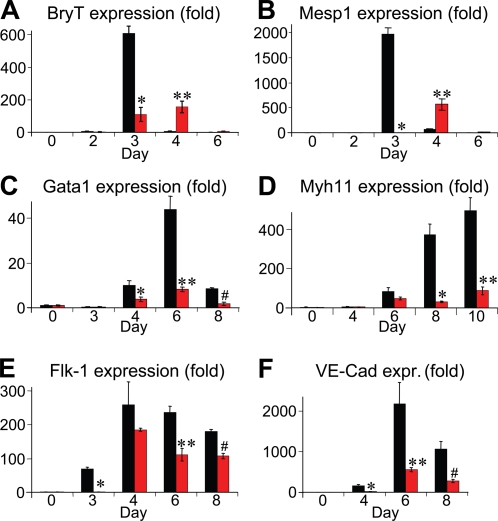
Dorsomorphin treatment promotes cardiomyogenesis at the expense of other mesodermal lineages. (A) Dorsomorphin treatment during the first 24 hours of ES cell differentiation (from day 0 to 1) blunted the induction of BryT expression at day 3 (*p = 0.0016), but resulted in higher BryT expression at day 4, in comparison to controls (**p = 0.017). (B) Dorsomorphin treatment caused similar, yet even more striking, changes in Mesp1 expression (*p<0.0001, **p = 0.0107). In addition, dorsomorphin treatment resulted in significant decreases in (C) Gata1 expression at days 4 to 8 (*p = 0.0382, **p = 0.0044, and #p = 0.0019), in (D) MyH11 expression at days 8 and 10 (*p = 0.0032 and **p = 0.0040), in (E) Flk-1 expression at day 3 to 6 (*p = 0.0002, **p = 0.0092, and #p = 0.0021), and in (F) VE-Cadherin (VE-cad; vascular endothelium-cadherin) expression at day 4 to 8 (*P = 0.0076, **p = 0.0364, and #P = 0.0153). All results are compared to DMSO control. Red bars, dorsomorphin-treated. Black bars, DMSO-treated. Q-PCR results were obtained from at least three independent experiments. Error bars, standard error.

To gain insight into how dorsomorphin treatment promotes cardiomyogenesis, we examined expression of several markers of mesoderm-derived lineages in ES cells treated with 2 µM DM at day 0 to 1 and compared them to DMSO-treated cells. In contrast to the earlier finding with Noggin, which dramatically increased expression of the mesoderm marker Brachyury T (BryT) at day 3, dorsomorphin treatment (day 0 to 1) significantly reduced BryT expression at day 3 (**Figure 5A**). However, with dorsomorphin treatment, significant levels of BryT expression persisted to day 4, when BryT expression was extinguished in controls (**Figure 5A**). Dorsomorphin treatment caused even more striking changes to the expression of Mesp1, one of the earliest known markers of cardiac progenitor cells [22]. With dorsomorphin treatment, onset of significant Mesp1 expression did not occur until day 4, one day later and at moderately lower levels than in controls (**Figure 5B**). Thus, in contrast to Noggin treatment or other reported manipulations that promote cardiomyogenesis in ES cells [10]–[12], [23], dorsomorphin treatment does not simply increase peak expression of mesoderm makers per se, but rather delays the onset of their expression.

To examine how changes in the expression profiles of early mesodermal markers affect the differentiation of other lineages of mesodermal origin, we analyzed the timing and quantified the levels of expression of blood, endothelial and smooth muscle cell-specific markers. In contrast to cardiomyocyte markers, we found that dorsomorphin treatment from day 0 to 1 decreased the expression of the hematopoietic progenitor marker Gata1 (**Figure 5C**) and the smooth muscle-specific myosin heavy chain gene Myh11 (**Figure 5D**). In addition, dorsomorphin treatment led to sustained decreases in the expression of the vascular marker Flk-1 (Vegfr2) from day 3 to 8 (**Figure 5E**). Whereas Flk-1 expression is not endothelium-restricted [14], this result, together with the reduced vascular endothelial-cadherin (VE-Cad) expression at days 6 and 8 (**Figure 5F**), suggests that dorsomorphin treatment decreases endothelial cell differentiation. Similar results were seen for day −3 to 2 dorsomorphin treatment (**Figure S1A to E**). Thus, pharmacological blockade of BMP signaling during the initial stages of ES cell differentiation appears to promote formation of pre-cardiac mesodermal cells at the expense of endothelial, smooth muscle and hematopoietic lineages.

## Discussion

Small molecules that selectively modulate developmental pathways hold promise as versatile tools for dissecting signaling pathways involved in lineage commitment of pluripotent stem cells and for directing stem cell differentiation toward desired cell types [11], [12]. Here, we have used dorsomorphin, a recently described small molecule inhibitor of BMP signaling, to reproducibly and substantially induce cardiomyogenesis in mouse ES cells. Dorsomorphin treatment during the first 24 hours of differentiation was sufficient for robust cardiac induction at the expense of other mesoderm-derived lineages.

Our findings are generally in line with those obtained using the endogenous BMP antagonist Noggin [10]; however, several important differences are worth noting. First, whereas the Noggin application must begin prior to the initiation of EB formation for a total duration of 5 days to achieve efficient cardiomyogenesis, dorsomorphin treatment begun at the time of EB formation and continued for just 24 hours was able to induce cardiomyogenesis very efficiently. Second, in contrast to Noggin, dorsomorphin treatment did not increase the peak expression of the early mesoderm marker BryT at day 3 of differentiation, but rather delayed the temporal pattern of its expression. Dorsomorphin treatment also led to a delay in the onset of the cardiac progenitor marker Mesp1 expression.

It is possible that the overall duration and later persistence of BryT and Mesp1 expression, not their transient peak levels, better reflect the number of BryT+ or Mesp1+ cells generated. This, together with the fact that dorsomorphin treatment limited to the initial 24 hours of differentiation was sufficient for robust cardiac induction, lead us to propose that dorsomorphin acts on a very primitive pluripotent cell type to promote the cardiomyogenic program.

Together with increases in the expression of cardiac-specific genes and decreases in the expression of endothelial, smooth muscle and hematopoietic markers, the dorsomorphin-induced delay in the temporal expression patterns of BryT and Mesp1 is consistent with a substantial shift in the developmental repertoire of mesodermal cells toward the formation of cardiac precursor cells, which arise subsequent to the formation of the hemangioblastic population [8], [14]. Based on the prior report with Noggin [10], we hypothesize that the dorsomorphin effect is cell-autonomous, acting directly on primitive pluripotent cells to increase the proportion that becomes committed to the cardiac lineage, consequently leaving less for endothelial, smooth muscle and hematopoietic development. Although the detailed mechanism by which dorsomorphin promotes the formation of committed cardiac precursors is yet to be determined, our findings provide additional support for the interconnection between the myocardial and the noncardiac mesodermal lineages as they diverge from a common progenitor [14], [25], [27]–[30], and suggest the critical role of BMP signaling in regulating this process.

Distinct effects of Noggin and dorsomorphin on ES cell differentiation may reflect intrinsic differences between the small molecule dorsomorphin and protein-based BMP antagonists. Dorsomorphin efficiently induces cardiomyogenesis when added at the onset of differentiation, whereas robust induction by Noggin is observed only when it is added prior to EB formation. This difference may reflect the small molecule's ability to readily penetrate multiple cell layers in developing EBs. In contrast, endogenous antagonists like Noggin may not gain full access to cells once the EB is formed. The differences could also arise from the fact that dorsomorphin appears to target multiple type-I BMP receptor subtypes [18], whereas Noggin's effects may be limited to antagonizing specific BMP ligands. Finally, a caveat to consider with a small molecule like dorsomorphin is the potential impact on off-target effects. For instance, the substantial reduction in peak BryT expression with dorsomorphin treatment could reflect the small molecule's effects on non-BMP signaling that negatively influence early mesoderm formation. Development of small molecules which are more selective for BMP signaling will be essential to clarify this issue.

In summary, we have utilized a selective small molecule inhibitor of BMP signaling, an important developmental pathway, to promote differentiation of primitive pluripotent cells toward cardiac cells. While it remains to be seen whether small molecule inhibitors of BMP signaling can also induce cardiac differentiation of other stem cell types, including the induced pluripotent stem (iPS) cells made from adult somatic tissue, the inherent advantages of small molecules like dorsomorphin could prove valuable for translation of recent stem cell advances into regenerative therapies. Dorsomorphin, which unlike endogenous BMP antagonists does not exhibit limited selectivity for ligand subtypes [20], can expand empiric efforts to modulate stem cell differentiation even in contexts where the specific cocktail of active BMPs is unknown. Moreover, precise temporal control afforded by a small molecule could prove to be critical for functional dissection of BMP signaling in complex biological contexts like organogenesis, where BMP pathway function at multiple developmental nodes with often divergent effects. Finally, because small molecules are relatively inexpensive, can penetrate many cell layers, and may be orally bioavailable, pharmacologic modulators of key developmental pathways will prove to be useful, not just to control differentiation of pluripotent stem cells *in vitro*, but also to enhance the regenerative potential of resident stem cells *in vivo*.

## Materials and Methods

### Cell culture

Murine ES cell lines, CGR8 and R1, were grown in feeder-free conditions as monolayers. The CGR8 ES cell line was transfected with the nuclear-localized red fluorescent protein (DsRed-Nuc) gene that is expressed under the cardiac α-myosin heavy chain promoter (the α-MHC promoter vector was kindly provided by J. Robbins and M. Anderson, and pDsRed-Nuc vector was purchased from Clontech). CGR8 cells were maintained in GMEM (Sigma-Aldrich) supplemented with 10% FBS (Gibco), 2 mM l-glutamine, (Cellgro), 0.05 mM 2-mercaptoethanol (Sigma-Aldrich), and 200 U/ml murine LIF (Chemicon International). R1 cells were maintained in High Glucose DMEM (Gibco) supplemented with 15% FBS, 2 mM l-glutamine, 1× nonessential amino acids, 100 U/ml penicillin-100 µg/ml streptomycin (Cellgro), 0.05 mM 2-mercaptoethanol, 1 mM sodium pyruvate (Sigma-Aldrich), and 200 U/ml murine LIF. Both cell lines were cultured on 0.2% gelatin-coated dishes. Every 24 hours, cells were washed in 1× PBS and culture media was replaced. Cells were passaged when confluence reached 50–60% to preserve the undifferentiated phenotype.

### Initiation of Dorsomorphin Treatment

For experiments in which dorsomorphin treatment was begun prior to EB formation, ES cell cultures at 10% confluence were treated with ES media supplemented with 2 µM dorsomorphin (Compound C, Sigma-Aldrich) dissolved in DMSO (Sigma-Aldrich). Cells treated with media containing an equivalent amount of DMSO served as a negative control. ES media containing either dorsomorphin or DMSO was changed daily for three days. DMSO-treated cells had similar outcomes to control untreated cells, indicating that DMSO had no effect on ES cell growth and differentiation.

### ES cell Differentiation

After ES cell treatment with dorsomorphin or DMSO for three days (on Day 0), ES cells were trypsinized and embryoid bodies (EBs) were generated by the three-dimensional hanging drop method (Day 0). Briefly, EBs were grown in hanging drops for two days (Day 0 to Day 2), each of which initially consisted of 500 cells in 19 µL of EB differentiation media. The EB differentiation media was composed of IMDM (Gibco) supplemented with 20% FBS, 1.6 mM l-glutamine, 1× nonessential amino acids, 0.08 mM 2-mercaptoethanol, and either 2 µM dorsomorphin or DMSO. For R1 cells, differentiation media additionally contained 1 mM sodium pyruvate. At day 2 of differentiation (Day 2), treatment with dorsomorphin or DMSO was discontinued. The EBs were transferred to uncoated Petri dishes and suspended in differentiation media for two days (Day 2–Day 4). On Day 4, the EBs were moved to gelatin-coated 6-well plates, allowed to attach and incubated in differentiation media until Day 14. In certain experiments, differentiated cells were kept in culture for several weeks for observation of long-term effects. Throughout this time, the media was replaced every 48–72 hours. Each day, differentiating cell clusters were microscopically examined for the presence of contracting cardiomyocytes and, in the case of engineer CGR8 cells, red fluorescence.

A second culture technique was used to form embryoid bodies, which allowed us to quantify the number of contracting EBs. The ES cells were grown in accordance with the aforementioned methods. Rather than constructing hanging drops on day 0, aliquots of cells were distributed in uncoated 96-well round bottom plates, and 100 µL of dorsomorphin- or DMSO-containing differentiation media was added to each well. Beginning on day 2, the media was replaced every 48–72 hours with differentiation media lacking dorsomorphin or DMSO. EBs were microscopically examined for contracting cardiomyocytes on days 8 through 12. Any well containing spontaneously beating cells was recorded as 1 positive result.

For experiments in which dorsomorphin treatment was begun at the time of EB formation, EBs were generated by the aforementioned methods in the presence of dorsomorphin or DMSO. At specified times, treatment with dorsomorphin or DMSO was discontinued.

### Quantification of DsRed+ cells

Embryoid bodies (EB) were grown on gelatin-coated plates for 12 days following dorsomorphin or DMSO treatment. On day 12, EBs expressing DsRed-Nuc under the α-MHC promoter were dissociated with trypsin treatment, stained with 4′-6-Diamidino-2-phenylindole (DAPI), and 2 µL aliquots were placed on cover slips to count the DsRed+ nuclei and the total DAPI stained nuclei under a fluorescence microscope.

### FACS analysis

EBs were dissociated into single cell suspensions after trypsinization. Following a wash with 10%FBS/DMEM, cells were permeabilized with 0.05% saponin/PBS buffer for 20 minutes on ice. Cells were then stained with an α-Actinin antibody (Sigma; 1∶100dilution in 10%FBS/DMEM) for 1 hour. Following washes with 10%FBS/DMEM, cells were incubated with an anti-mouse secondary antibody conjugated to AlexaFluor-488 (1∶200 dilution in 10%FBS/DMEM) for 30 minutes in dark. After additional washes in 10%FBS/DMEM, cells were resuspended in 300 µL 10%FBS/DMEM and analyzed on the 5-laser BD LSRII FACS instrument.

### Immunostaining and confocal microscopy

EBs treated with dorsomorphin or DMSO (day 0∼1) were plated at day 4 on glass cover slip culture chambers coated with 1% gelatin. At day 10, EBs were fixed in 5% formaldehyde at room temperature for 30 minutes, and then permeabilized with 0.2% Triton X-100 in PBS. After blocking with 1 mg/ml BSA in PBS, cells were incubated with mouse monoclonal anti-α-Actinin (Sigma), mouse cardiac alpha-myosin heavy chain (Abcam), or mouse cardiac Troponin T (Santa Cruz) antibodies along with goat Nkx2.5 antibody (Santa Cruz) at concentrations recommended by the manufacturers. After overnight incubation, cells were washed several times with PBS and then incubated with the AlexaFluor-488-conjugated rabbit anti-mouse IgG (Molecular Probes) and Cy3-conjugated-AffiniPure rabbit anti-goat IgG (Jackson Immuno). Immunostaining images were obtained using both a Leica inverted microscope (10×) and a Zeiss inverted LSM 510 confocal microscope (40×).

### Quantitative real-time PCR

Cells were harvested on days 0, 2, 3, 4, 6, 8, 10, 12 of EB differentiation and stored at −80°C in cell lysis buffer RLT (Qiagen). Three independent samples were collected for each time point studied. Total RNA was extracted using the RNeasy Mini Kit according to the manufacturer's instructions and treated with RNase-free DNase I (Qiagen). First-strand cDNA was synthesized with the SuperScript III First-Strand Synthesis SuperMix for qRT-PCR (Invitrogen). Using cDNA as template, TaqMan real-time PCR assays was performed in triplicates on the ABI Prism 7900 HT sequence detection system (Applied Biosystems) according to the manufacturer's instructions. Data were normalized to GAPDH, and levels of gene expression were normalized to that of Day 0 DMSO-treated cells. The following TaqMan probe and primer sets (Applied Biosystems) were used: Id1 (Mm00775963_g1), nkx2.5 (Mm00657783_m1), myh6 (Mm00440354_m1), myl2 (Mm00440384_m1), brachyury T (Mm00436877_m1), flk-1 (Mm00440099_m1), myh11 (Mm00443013_m1), gata1 (Mm00484678_m1), ve-cadherin (Mm00486938_m1), mesp1 (Mm00801883_g1), and GAPDH (Mm99999915_g1).

### Western Blotting

Lysates of EBs on day 10 were separated by SDS/PAGE and transferred onto PVDF membrane. The α-MHC protein was detected by Odyssey system (Li-Cor bioscience) following incubation with mouse α-MHC antibody (Abcam, 1∶500 dilution) and IRDye 800CW-conjugated goat anti-mouse IgG (Li-Cor Bioscience, 1∶2000 dilution). Mouse α-tubulin antibody (Abcam, 1∶2000) was used as a loading control.

## Supporting Information

Figure S1 Dorsomorphin treatment from day −3 to 2 promotes cardiomyogenesis at the expense of other mesodermal lineages. (A) Dorsomorphin treatment (from day −3 to 2) blunted the induction of BryT expression at day 3 of differentiation (*p = 0.01), but resulted in higher BryT expression at day 4, in comparison to controls (**p = 0.0424). Dorsomorphin treatment resulted in significant decrease in (B) Flk-1 expression at day 3 to 6 (*p = 0.0353 and #p = 0.0237), in (C) VE-Cadherin (VE-cad; vascular endothelium-cadherin) expression at day 4 and 6 (*P = 0.0042 and **P = 0.0018), in (D) MyH11 expression at days 8 and 10 (*p = 0.0104 and #p<0.0001), and in (E) Gata1 expression at days 4, 6 and 8 (*p = 0.0016, **p = 0.0003 and #p = 0.0452). (F) Dorsomorphin treatment (day −3 to 2) increased cardiac myosin light chain 2 (Myl2) expression at day 10 by 34.2-fold over control. All results compared to DMSO control. Red bars, dorsomorphin-treated. Black bars, DMSO-treated. Q-PCR results, except for Myl2, were obtained from at least three independent experiments. Error bars, standard error.
(0.65 MB TIF)

Figure S2 Dorsomorphin (DM) treatment of ES cells efficiently blocks activation of the BMP-target gene Id1. Treatment of mouse ES cells with 2 µM DM from day 0 to 1 of differentiation resulted in a 96.1% reduction in the expression levels of the BMP-target gene Id1 at day 1 (*p<0.0001). All results are compared to DMSO-vehicle treatment as negative control. Red bars, dorsomorphin-treated. Black bars, DMSO-treated. Error bars represent standard error. Q-PCR results represent relative expression normalized to that of DMSO-treated cells at day 0. Results were obtained from at least three independent experiments.
(0.34 MB TIF)

Movie S1 Spontaneously contracting focus of cardiomyocytes expressing DsRed-Nuc protein under the α-MHC promoter formed from dorsomorphin-treated ES cells at day 12 of differentiation.
(1.15 MB AVI)

Movie S2 Phase contrast movie of spontaneously contracting foci of cardiomyocytes formed from dorsomorphin-treated ES cells on a gelatin-coated plate.
(0.89 MB AVI)

Movie S3 Spontaneously contracting EB formed from dorsomorphin-treated ES cells on a 96-well microtiter plate.
(0.81 MB AVI)
