# Supplementary figures and images for: Dorsomorphin, a Selective Small Molecule Inhibitor of BMP Signaling, Promotes Cardiomyogenesis in Embryonic Stem Cells
Source: PLoS One. 2008 Aug 6;3(8):e2904. doi: 10.1371/journal.pone.0002904 (PMC2483414; doi:10.1371/journal.pone.0002904)

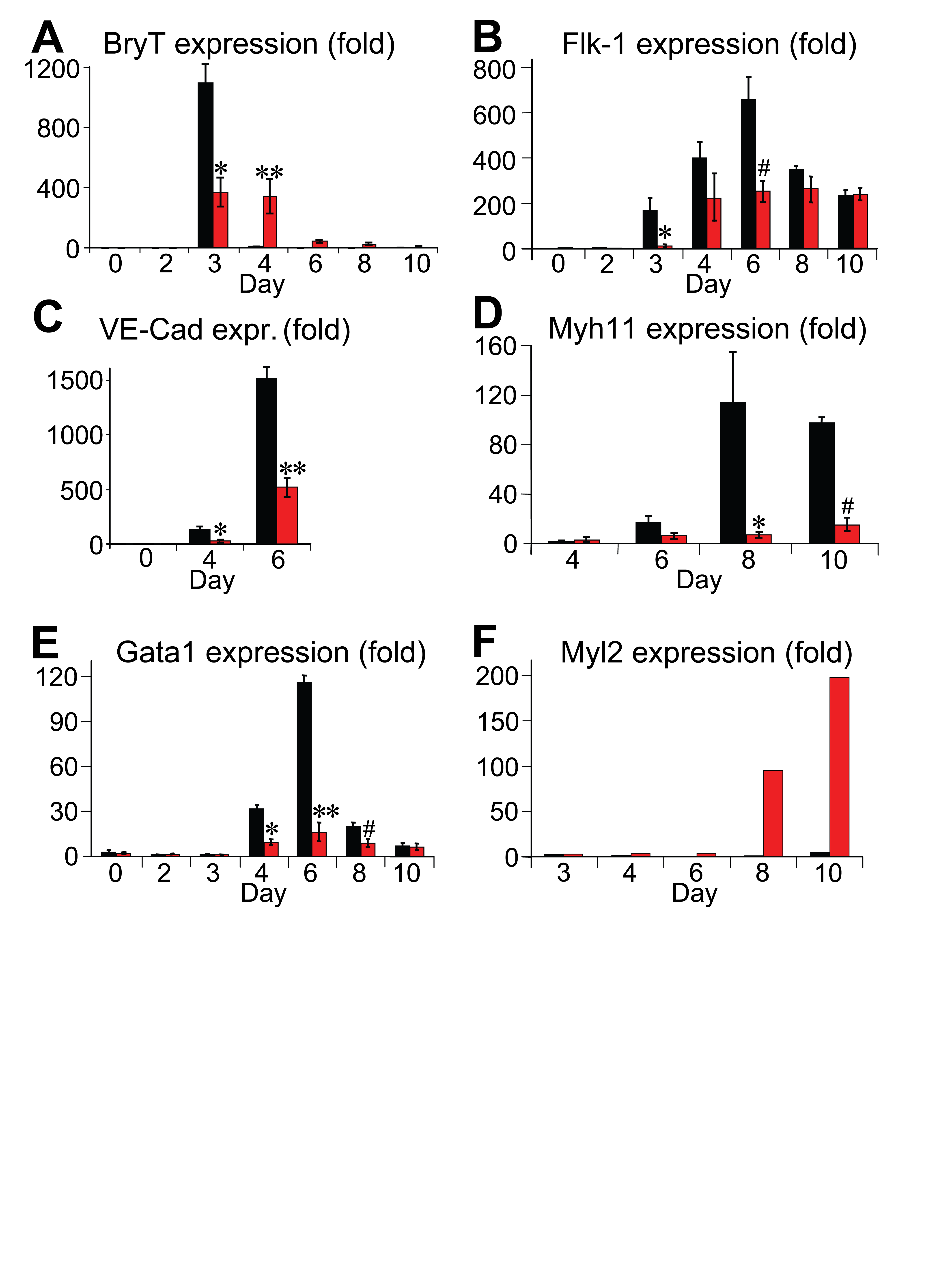

Supplement: Figure S1 — Dorsomorphin treatment from day −3 to 2 promotes cardiomyogenesis at the expense of other mesodermal lineages. (A) Dorsomorphin treatment (from day −3 to 2) blunted the induction of BryT expression at day 3 of differentiation (*p = 0.01), but resulted in higher BryT expression at day 4, in comparison to controls (**p = 0.0424). Dorsomorphin treatment resulted in significant decrease in (B) Flk-1 expression at day 3 to 6 (*p = 0.0353 and #p = 0.0237), in (C) VE-Cadherin (VE-cad; vascular endothelium-cadherin) expression at day 4 and 6 (*P = 0.0042 and **P = 0.0018), in (D) MyH11 expression at days 8 and 10 (*p = 0.0104 and #p<0.0001), and in (E) Gata1 expression at days 4, 6 and 8 (*p = 0.0016, **p = 0.0003 and #p = 0.0452). (F) Dorsomorphin treatment (day −3 to 2) increased cardiac myosin light chain 2 (Myl2) expression at day 10 by 34.2-fold over control. All results compared to DMSO control. Red bars, dorsomorphin-treated. Black bars, DMSO-treated. Q-PCR results, except for Myl2, were obtained from at least three independent experiments. Error bars, standard error. (0.65 MB TIF) [file pone.0002904.s001.tif]

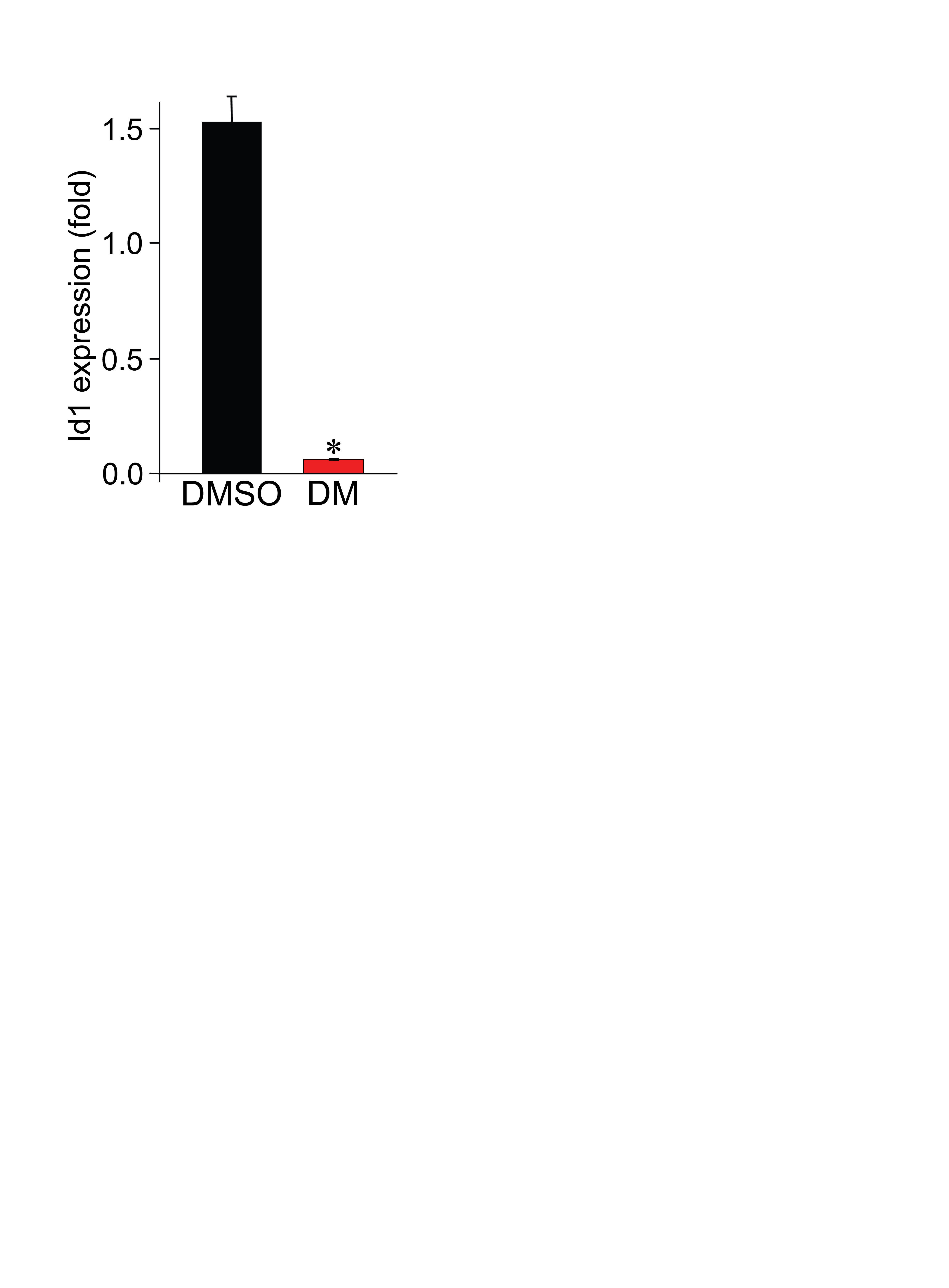

Supplement: Figure S2 — Dorsomorphin (DM) treatment of ES cells efficiently blocks activation of the BMP-target gene Id1. Treatment of mouse ES cells with 2 µM DM from day 0 to 1 of differentiation resulted in a 96.1% reduction in the expression levels of the BMP-target gene Id1 at day 1 (*p<0.0001). All results are compared to DMSO-vehicle treatment as negative control. Red bars, dorsomorphin-treated. Black bars, DMSO-treated. Error bars represent standard error. Q-PCR results represent relative expression normalized to that of DMSO-treated cells at day 0. Results were obtained from at least three independent experiments. (0.34 MB TIF) [file pone.0002904.s002.tif]
